# Outcomes associated with observation versus short-stay admission among chest pain patients in the Veterans Health Administration

**DOI:** 10.1186/s12873-016-0103-4

**Published:** 2016-09-21

**Authors:** Brad Wright, Amy M. J. O’Shea, Justin M. Glasgow, Padmaja Ayyagari, Mary Vaughan-Sarrazin

**Affiliations:** 1 Department of Health Management and Policy, College of Public Health, University of Iowa, 145 North Riverside Drive, N240 CPHB, Iowa City, IA 52242 USA; 2 Public Policy Center, University of Iowa, Iowa City, IA USA; 3 Iowa City Veterans Affairs Healthcare System, The Comprehensive Access and Delivery, Research and Evaluation Center (CADRE), Iowa City, IA 52242 USA; 4 Department of Internal Medicine, Carver College of Medicine, University of Iowa, Iowa City, IA 52242 USA; 5 Christiana Care Health System Department of Internal Medicine and Christiana Care Health System Value Institute, Wilmington, DE USA

**Keywords:** Observation stays, Short-stay admissions, Readmissions, Mortality, Veterans Health Administration

## Abstract

**Background:**

To determine the extent to which 30- and 90-day hospital readmission and mortality rates differ as a function of whether a chest pain patient is placed in observation status or admitted to the hospital for a short-stay (<48 h).

**Methods:**

Using 114,043 observation stays and short-stay admissions for chest pain at Veterans Health Administration hospitals between 2005 and 2013, we estimated event-level logistic regression models using a generalized estimating equation framework to predict 30 and 90-day readmissions and mortality as a function of whether the patient had an observation stay or a short-stay admission. We also adjusted for a variety of patient characteristics and unobserved time-invariant hospital factors.

**Results:**

Relative to the short-stay inpatient group, veterans with chest pain who were placed in observation status were significantly more likely to be female (7.0 % vs. 6.4 %, White (76.6 % vs. 71.0 %, and from a rural area (28.3 % vs. 20.2 %). There were no other meaningful differences between the groups. Veterans with chest pain who were placed in observation status had 25 % lower odds of dying within 30 days (95 % confidence interval [CI]: 3 % – 43 %) and 12 % lower odds of a 30-day readmission (95 % CI: 6 % – 17 %) compared to those admitted as short-stay inpatients. Neither 90-day outcome was significantly associated with placement in observation status. Patient demographics were also important predictors of mortality and readmissions.

**Conclusions:**

There are clinically observable differences in outcomes between patients admitted to observation and those admitted as short-stay inpatients. We find no evidence that the increase in observation stays reflects a lack of proper care for patients placed in observation status.

## Background

Patients who cannot be safely discharged home from the emergency department, but who are not necessarily ill enough to be admitted to the hospital as inpatients are often placed in observation status. Patients in observation status may receive care in a dedicated observation unit which may or may not be protocol-driven, or they may simply be placed in a bed on a general ward where they receive care that may or may not be protocol-driven with the expectation that they will be able to go home relatively soon. In other words, there is variation in the extent to which being placed in observation status denotes a particular type of care, or is merely a billing code [1, 2]. While there are well established clinical benefits to protocol-driven observation care, [1] critics have described observation stays as “a modern-day purgatory” because of the ambiguity surrounding their use [3].

In the Veterans Health Administration (VHA), observation stays have historically represented a difference in billing but not in placement or care management. That is, the VHA does not operate dedicated observation units, and does not explicitly deliver protocol-driven observation care. If the only distinction in the VHA between a short-stay inpatient admission (less than 48 h) and an observation stay is a billing code (i.e., the patient is in the same bed on the same floor receiving the same services), it raises the question of why the observation code is necessary.

However, prior research has demonstrated that observation in the VHA not only persists, but also happens to be increasing rapidly. Indeed, the rate of observation stays in the VHA has more than doubled between 2005 and 2013, although the rates vary significantly across hospitals [4]. This is similar to trends among Medicare beneficiaries [5]. Recent research by our team has identified both hospital-level factors that explain that variation, and patient characteristics associated with a greater likelihood of being placed in observation status rather than being admitted for a short-stay [6]. Yet, the clinical consequences of the increase in the observation stay rate and its variation across and within hospitals are unknown, and there is conflicting evidence as to whether observation stay patients are clinically distinct from individuals admitted for short stays as inpatients [7, 8].

This study aims to answer the question: To what extent do patient outcomes differ depending on whether a patient is placed in observation status or admitted for a short-stay? Specifically, we model 30- and 90-day hospital readmission and mortality rates for chest pain patients as a function of whether they were placed in observation status or admitted to the hospital for a short-stay of less than 48 h.

## Methods

### Study design and data source

Our longitudinal observational study was based on a combined sample of more than 114,000 observation stays and short-stay admissions (<48 h) with a diagnosis of chest pain at VHA hospitals identified from 2005 to 2013 in the VHA Medical SAS inpatient files. The selection of our analytic sample from the more than 4.4 million total acute admission records in the VHA during those nine years has been previously described elsewhere [6]. We merged these records with patient demographic data available in corresponding years of the VHA enrollment file. We limited our sample to individuals with a clinical classification software (CCS) diagnosis of chest pain to ensure comparability between groups. CCS diagnoses rely on ICD-9 diagnosis codes, collapsing them to a smaller and more intuitively meaningful number of conditions. We selected chest pain because it is one of the most common presentations in both settings and is well-suited to evaluation and management protocols under observation status.

### Key independent and outcome variables

Our key independent variable was a binary measure indicating whether or not a patient had an observation stay. The variable was equal to one if the patient was initially placed in observation (regardless of subsequent inpatient admission, which occurred in 26.2 % of observation stays), and zero if the patient was initially admitted as an inpatient for a short-stay, which we defined as 48 h or less in duration. To ensure that short-stays did not include stays that would potentially have been longer and that mortality rates for either observation stays or short-stays were not influenced by differential rates of death within the hospital, we excluded individuals who died in the hospital (*n* = 11) or who were transferred out of the hospital, rather than being discharged home. These events were identified using VHA bedsection codes, and we excluded all veterans from our sample who did not have any observation stays or short-stay admissions in a given year.

Our primary outcomes of interest in this study were 30-day and 90-day readmissions and 30-day and 90-day post-discharge mortality rates. Using each healthcare event, whether an observation stay or short-stay admission, we looked forward 30 days and 90 days from the current discharge date. If the patient experienced at least one other admission of any duration at any VHA hospital within those time frames, we flagged them as having a 30-day or 90-day readmission, respectively. Thus, readmissions may include observation stays, short-stay inpatient admissions, and longer inpatient admissions. Similarly, if the VHA enrollment file indicated that the patient died within 30 or 90 days from the current discharge date, we flagged them as 30-day or 90-day mortality, respectively.

### Analysis

We used a generalized estimating equation (GEE) framework with an exchangeable working correlation structure to estimate an event-level logistic regression model predicting patient outcomes as a function of whether or not the patient had an observation stay or a short-stay admission. The GEE method, in conjunction with robust standard errors clustered at the patient level, accounted for the fact that people could have more than one hospital visit each year and that data would likely be correlated across these visits. We estimated separate models for each of our four outcomes. All analyses were conducted using SAS.

In addition to our key independent variable, we controlled for several potential confounding variables, including patient age (in years), gender, race/ethnicity, rurality of residence, homelessness, copayment status, and number of comorbid conditions. We identified comorbid conditions using ICD-9-CM diagnosis codes and methods first developed by Charlson and subsequently updated by Quan and colleagues [9]. To control for other unobserved factors that might be associated with our outcomes of interest over time, we included a series of year dummy variables. Finally, to account for unobserved time-invariant hospital factors that might be associated with readmission and mortality rates, we specified 102 hospital-specific fixed effects in the model. For all models, 40 VHA hospitals were dropped from our sample because of perfect prediction (i.e., they had no within-hospital variation in the outcomes of interest, making it impossible to model the outcome since we included hospital fixed effects).

## Results

### Characteristics of study subjects

Out of 114,043 patient events across 102 hospitals in our sample, nearly 37.5 % were observation stays, while 62.5 % were short inpatient stays. A total of 26.2 % of the observation stay events progressed to an inpatient admission, but were treated as observation stays for the purposes of our analyses. Complete descriptive statistics of the veterans in our sample, broken out by observation stays versus short-stay inpatient admissions are shown in Table 1. While almost all of the differences in observable characteristics between the two groups are statistically significant, there do not appear to be clinically meaningful differences between them. The average veteran in both groups of our sample was a white male, just over 62 years old, living in an urban area, with an average of 1.5 to 1.6 comorbid conditions. The average length of stay for observation stay patients (excluding the inpatient portion of the stay among those whose status converted) was just less than a day, while the average length of stay for short-stay patients (whose stays were truncated at 48 h by definition) was just over 1 day. This average individual was subject to making full copayments for their care in the VHA, and was highly unlikely to be homeless.

**Table 1:** Descriptive statistics by type of hospital stay

Variable	Observation stay	Short-stay inpatient	*p*-value
Age in years (SD)	62.2 (12.1)	62.3 (11.9)	0.1851
Female (%)	7.0	6.4	<0.0001
Race (%)			
Asian	0.8	1.0	<0.0001
Black	19.4	23.0	
Hispanic	2.4	4.1	
Native American	0.8	0.8	
White	76.6	71.0	
Homeless (%)	1.1	1.2	0.0325
Count of comorbidities (SD)	1.5 (2.2)	1.6 (2.2)	<0.0001
Rurality of residence (%)			
Isolated	7.0	5.1	<0.0001
Small rural	8.4	5.7	
Large rural	12.9	9.5	
Urban	71.7	79.8	
Copayment category (%)			
No copayment	9.5	9.2	<0.0001
Reduced copayment	1.6	2.0	
Full copayment	89.0	88.9	
Length of stay in days (SD)	0.86 (0.56)	1.12 (0.45)	<0.0001
Fiscal year (%)			
2005	7.1	11.2	<0.0001
2006	7.7	11.2	
2007	8.1	11.5	
2008	8.3	11.8	
2009	9.6	12.1	
2010	11.6	12.5	
2011	13.4	11.0	
2012	17.0	10.0	
2013	17.2	8.7	
Sample size	*N* = 42,704	*N* = 71,339	

*SD* standard deviation

### Mortality results

The results of our models to predict 30-day and 90-day post-discharge mortality are shown in Table 2. Of primary interest, we find that among our sample of chest pain patients, having been placed in observation status rather than admitted for a short-stay was associated with a lower likelihood of death that appears to diminish over time post-discharge. Specifically, those veterans placed in observation status had 25 % lower odds of dying within 30 days after discharge than those with a short-stay admission. However, when the follow-up window was expanded to 90-days post-discharge, the relationship between observation status and the odds of dying was no longer significant.

**Table 2:** 30- and 90-day mortality

Variable	30-day mortality		90-day mortality	
	OR	95 % CI	OR	95 % CI
Observation stay	0.75	[0.57, 0.97]	0.90	[0.77, 1.06]
Age	1.06	[1.05, 1.07]	1.06	[1.05, 1.07]
Female (vs. male)	0.23	[0.09, 0.62]	0.34	[0.21, 0.56]
Race (vs. white)				
Asian	1.09	[0.41, 2.92]	0.99	[0.53, 1.87]
Black	0.76	[0.55, 1.03]	0.89	[0.74, 1.06]
Hispanic	0.83	[0.42, 1.64]	0.66	[0.43, 1.04]
Native American	1.39	[0.52, 3.68]	1.52	[0.86, 2.69]
Homeless (vs. not)	1.39	[0.51, 3.82]	2.23	[1.34, 3.71]
Count of comorbidities	1.06	[1.01, 1.12]	1.05	[1.02, 1.08]
Rurality of residence (vs. urban)				
Isolated	0.66	[0.40, 1.08]	0.87	[0.66, 1.15]
Small rural	0.86	[0.55, 1.34]	0.89	[0.68, 1.18]
Large rural	1.02	[0.72, 1.43]	1.06	[0.85, 1.32]
Copayment category (vs. reduced)				
No copayment	0.64	[0.31, 1.31]	0.58	[0.37, 0.91]
Full copayment	0.70	[0.37, 1.32]	0.75	[0.50, 1.11]
Fiscal year (vs. 2005)				
2006	0.54	[0.32, 0.89]	0.52	[0.38, 0.71]
2007	0.71	[0.45, 1.13]	0.70	[0.53, 0.92]
2008	0.83	[0.53, 1.30]	0.92	[0.71, 1.20]
2009	1.13	[0.75, 1.70]	0.98	[0.76, 1.26]
2010	0.93	[0.60, 1.43]	0.91	[0.70, 1.17]
2011	1.03	[0.66, 1.59]	0.94	[0.73, 1.22]
2012	0.72	[0.46, 1.13]	0.82	[0.63, 1.06]
2013	0.99	[0.64, 1.52]	0.92	[0.71, 1.19]
	*N* = 114,043			

Patient demographics were also important predictors of mortality. Significantly reduced odds of mortality were observed among women, while increased odds of mortality were observed among those who were older, homeless, and/or those with a greater number of comorbid conditions. Specifically, women had 77 % lower odds of 30-day mortality and 66 % lower odds of 90-day mortality compared to men. By contrast, each additional year of age was associated with a 6 % increase in the odds of death post-discharge, and this relationship was the same for both 30-day and 90-day mortality models. While the homeless were not significantly more likely than the non-homeless to die within 30-days of discharge, they did have 123 % higher odds of dying within 90-days of discharge. Finally, the number of comorbid conditions was positively related to the likelihood of death within both 30-days and 90-days post-discharge. Each additional comorbid condition was associated with a 6 % increase in the odds of dying within 30-days, and a 5 % increase in the odds of dying within 90-days.

We found no relationship between patient race/ethnicity or rurality of residence and the likelihood of dying within 30 or 90 days after being discharged with chest pain. We also found no trend in the likelihood of 30- and 90-day post-discharge mortality among chest pain patients over time. Although we do not report the individual odds ratios for specific VHA hospitals, these fixed effects were jointly significant (*p* < 0.0001).

### Readmission results

The results of our models to predict 30-day and 90-day post-discharge readmission (which includes both observation and short and long inpatient stays) are shown in Table 3. Here we found that being placed in observation status was associated with 12 % lower odds of 30-day readmission compared to having been admitted for a short-stay. However, being placed in observation status was not significantly associated with the odds of a 90-day readmission compared to those admitted for a short-stay.

**Table 3:** 30- and 90-day readmission

Variable	30-day readmission		90-day readmission	
	OR	95 % CI	OR	95 % CI
Observation stay	0.88	[0.83, 0.94]	0.95	[0.90, 1.00]
Age	1.02	[1.02, 1.02]	1.02	[1.02, 1.02]
Female (vs. male)	0.72	[0.65, 0.80]	0.74	[0.68, 0.79]
Race (vs. white)				
Asian	0.71	[0.55, 0.92]	0.76	[0.62, 0.93]
Black	0.92	[0.86, 0.98]	0.94	[0.89, 0.98]
Hispanic	0.78	[0.67, 0.89]	0.83	[0.75, 0.93]
Native American	0.82	[0.63, 1.07]	0.91	[0.73, 1.15]
Homeless (vs. not)	1.27	[1.03, 1.56]	1.45	[1.23, 1.72]
Count of Comorbidities	1.02	[1.01, 1.04]	1.02	[1.00, 1.03]
Rurality of Residence (vs. urban)				
Isolated	0.85	[0.76, 0.94]	0.84	[0.77, 0.91]
Small rural	0.91	[0.82, 0.99]	0.91	[0.84, 0.98]
Large rural	0.96	[0.88, 1.04]	0.89	[0.84, 0.96]
Copayment category (vs. reduced)				
No copayment	0.82	[0.68, 1.00]	0.87	[0.75, 1.02]
Full copayment	1.16	[0.97, 1.40]	1.26	[1.09, 1.44]
Fiscal year (vs. 2005)				
2006	1.03	[0.92, 1.15]	0.98	[0.89, 1.07]
2007	1.01	[0.90, 1.12]	0.98	[0.90, 1.07]
2008	1.02	[0.92, 1.14]	0.96	[0.88, 1.05]
2009	1.12	[1.01, 1.24]	1.04	[0.95, 1.13]
2010	1.01	[0.91, 1.12]	1.01	[0.93, 1.10]
2011	0.97	[0.87, 1.08]	0.93	[0.85, 1.02]
2012	1.10	[0.99, 1.21]	1.02	[0.94, 1.12]
2013	1.02	[0.91, 1.13]	0.87	[0.80, 0.95]
	*N* = 114,043			

Patient demographics were also important predictors of experiencing a readmission. Similar to the findings in the mortality models, women were less likely than men to be readmitted after being discharged. Women had 28 % lower odds of 30-day readmission and 26 % lower odds of 90-day readmission compared to men. Also similar to the mortality models, age, homelessness, and the number of comorbid conditions were all associated with an increased likelihood of readmission. Each additional year of age was associated with a 2 % increase in the odds of readmission within either 30 or 90 days. The homeless were much more likely than the non-homeless to be readmitted within 30 or 90-days of discharge. Specifically, their odds of being readmitted within 30-days were 27 % higher and their odds of being readmitted within 90-days were 45 % higher. Each additional comorbid condition was associated with a 2 % increase in the odds of being readmitted within either 30 or 90 days.

Unlike the mortality models, racial and ethnic minorities generally had lower odds of readmission post-discharge compared to whites when there were statistically significant differences. Specifically, Asians, blacks and Hispanics had 29, 8 and 22 % lower odds of 30-day readmission, respectively, compared to whites. Similar, but less pronounced associations were identified when extending the follow-up period to 90-days post-discharge. However, there was no significant difference in either outcome among Native Americans compared to whites.

Compared to urban residents, rural residents had lower odds of being readmitted both 30 and 90-days post-discharge. Depending on whether the individual resided in an isolated, small rural, or large rural area and whether the follow-up window was 30 or 90 days, the odds of being readmitted were anywhere from 9 % to 16 % lower than they were for urban residents. Compared to those veterans subject to a reduced copayment, those who were subject to full copayments were more likely to be readmitted in 90 days. Specifically, the full copayment group had 26 % higher odds of being readmitted within 90 days post-discharge.

Like our finding for mortality, we did not find a strong time trend in the likelihood of 30 or 90-day readmission over our study period. As with the mortality models, VHA hospital fixed effects were jointly significant (*p* < 0.0001).

### Limitations

Our study is subject to some limitations. First, while we found that observation stays are less likely to lead to readmission, we did not distinguish whether readmission events that did occur represented an additional observation stay, a short-stay admission, or a “long-stay” admission. This limits our ability to understand whether observation patients have recurrent observation stays for a potentially ambulatory sensitive condition or if their readmission represents a significant decompensation of the initial presenting condition that was inadequately treated during the first observation stay. Second, we are only able to capture admissions to a VHA hospital. So the reduced odds of admissions observed across certain groups, including women, certain racial/ethnic minorities, and those living in rural areas, may not truly reflect lower rates of readmission. Instead, it may be that these veterans seek care at the VHA for certain conditions and go elsewhere for their other care, or cannot always present to a VHA hospital when acutely sick. Finally, our findings are limited to the VHA and may not be generalizable to other patient populations and care settings.

## Discussion

We modeled the likelihood of mortality and readmission within 30 and 90 days post-discharge to determine if veterans who were placed in observation status experienced different outcomes than veterans admitted as inpatients whose hospitalization lasted for 48 h or less. We found that veterans placed in observation status had a lower likelihood of mortality within 30 but not 90 days. Understandably, as the window for mortality is expanded to 90-days post-discharge, death is less likely to be related to the index event in question. This suggests that while VHA physicians may not have been able to determine whose hospitalization was likely to be less than 48 h, they did successfully identify patients of lower acuity and admit them to observation status.

One of the concerns that motivated this study was that racial and ethnic minority patients who are placed in observation status might have worse outcomes than whites. However, the results from this study show exactly the opposite. There were no statistically significant differences in mortality rates (30 and 90 day) among racial and ethnic minorities compared to whites. At the same time, when there were statistically significant differences in readmission rates (30 and 90 day), the rates were lower among racial and ethnic minorities compared to whites. This raises the question of whether racial and ethnic minority veterans are less able to utilize outpatient services, and are instead presenting at the emergency department with conditions that could be treated at an ambulatory clinic, prompting emergency and inpatient physicians to monitor them in a hospital setting overnight, because of concerns about access to care.

Outside of increasing age, male gender, and count of comorbid conditions, being a homeless veteran was the only modeled characteristic associated with higher 90-day mortality rates. Additionally, these veterans had high 30 and 90-day readmission rates. So while we speculate that racial and ethnic minority veterans may use inpatient resources in ways that are inefficient and potentially preventable, even increased utilization of healthcare resources cannot overcome the impact of an unstable living environment on the morbidity and mortality of homeless veterans.

Fortunately, we do find that being placed in observation status is associated with decreased odds of a 30-day readmission. This suggests that the presenting problem has been adequately addressed during the initial presentation, whether by transition via discharge or admission for further management. One of the concerns that motivated this study was that recent increases in observation rates might represent overuse of observation stays and that these patients might have poorer outcomes due to receiving a lower intensity of services or having time pressure to finalize a disposition. Our findings suggest that this is not the case, and that patients placed in observation are no more likely to bounce back than those who were initially admitted. This finding only holds for the first 30-days as observation admissions are not significantly associated with 90-day readmissions.

Compared to veterans paying a reduced copayment, we found that veterans subject to full copayments had higher odds of readmission. This is unlikely to be a financially motivated difference. The VHA copayment for an observation stay is $50 and would be payable at each observation admission. The copayment for an inpatient stay is $1260 and would cover any additional hospitalizations within 90-days. As a capitated system, the VHA does not benefit from readmitting patients and even repeated observation stay copays would not impact a hospital budget. One consideration is that since veterans subject to full copayments are more likely to have additional sources of health coverage, they may have more fragmented care leading to poor care transitions and increased readmission rates.

Since the VHA did not utilize observation units or observation care pathways during our study period, our results show that there are clinically observable differences between patients admitted to observation versus those admitted as inpatients even if those inpatients end up with a hospitalization lasting less than 48 h. While the overall value of distinguishing between an admission and an observation stay cannot be answered with this analysis, these data suggest that defining all hospitalizations of less than 48 h as an observation stay would improperly lump separate patient groups into a single payment model.

## Conclusions

This study modeled 30-day and 90-day mortality and readmission rates in a cohort of veterans with hospitalizations less than 48 h and found that those initially admitted to observation had decreased rates of mortality and readmission within 30-days post-discharge. We found no evidence that the increasing trend in observation stays reflects a lack of proper care for patients placed in observation status, even in the absence of a dedicated observation care pathway.

## Abbreviations

CCS: Clinical classification software
GEE: Generalized estimating equation
VHA: Veterans Health Administration.
